# Supplementary material for: Tea Tree Essential Oil Kills Escherichia coli and Staphylococcus epidermidis Persisters
Source: Biomolecules. 2023 Sep 18;13(9):1404. doi: 10.3390/biom13091404 (PMC10526169; doi:10.3390/biom13091404)
Supplement: Supplementary file 1 [file biomolecules-13-01404-s001.zip › biomolecules-2545449-supplementary.pdf]

Supplementary Materials

Tea Tree Essential Oil Kills *Escherichia coli* and *Staphylococcus epidermidis* Persisters

In order to isolate persister cells, a bacterial population is treated with a greater than minimum inhibitory concentration (MIC) of antibiotic over a period of time to form a biphasic killing curve. The second part of the curve, where there is a plateau showing no more cell death, indicates the formation of persisters. Figure S1 shows the determination of antibiotic concentration and treatment time conditions to isolate *E. coli* and *S. epidermidis* persister cells. Final antibiotic concentrations and treatment times to isolate persisters for tea tree essential oil (TTO) experiments were chosen by selecting a concentration or time on the second part (plateau) of the killing curve. For *E. coli* (Figure S1a, b), an ofloxacin concentration of 5 µg/mL and a treatment time of 24 h were chosen. For *S. epidermidis* (Figures S1c, d), a ciprofloxacin concentration of 10 µg/mL and a treatment time of 48 h were chosen.

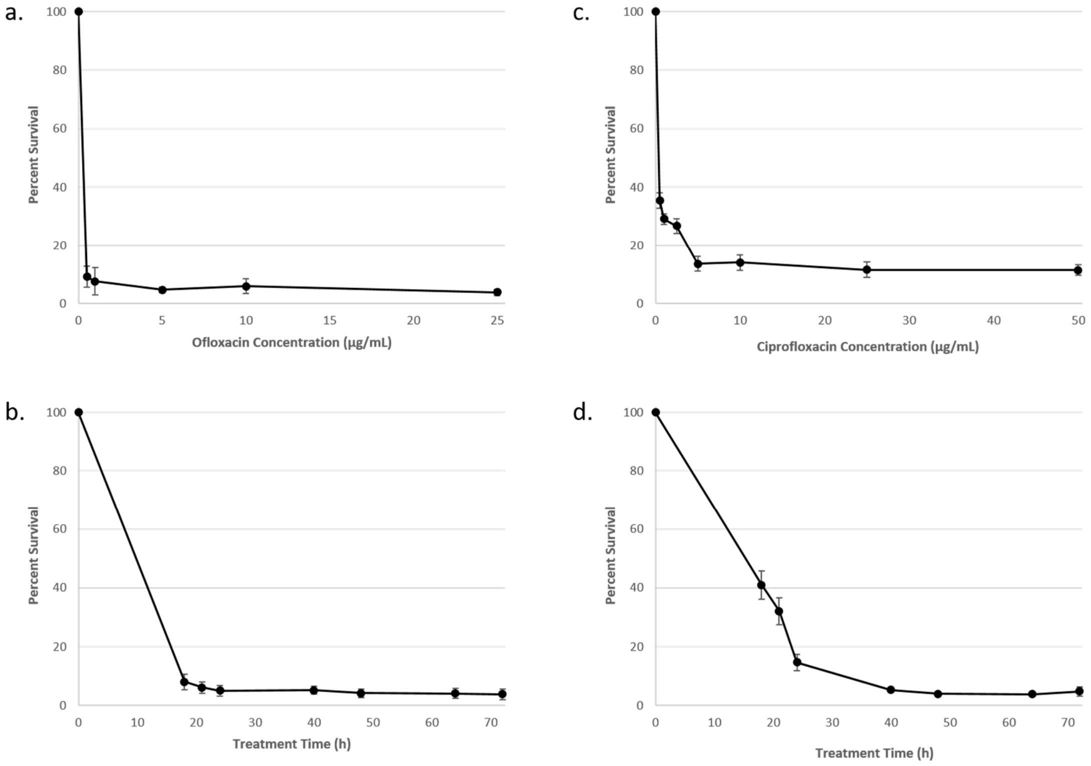

Figure S1. Determination of antibiotic concentration and treatment time conditions to isolate *E. coli* and *S. epidermidis* persister cells. Stationary phase *E. coli* culture was treated (**a**) with varying concentrations of ofloxacin over a 24 h period and (**b**) with 5 µg/mL of ofloxacin for varying times. Stationary phase *S. epidermidis* culture was treated (**c**) with varying concentrations of ciprofloxacin over a 24 h period and (**d**) with 10 µg/mL ciprofloxacin for varying times. At each time point, samples were removed, centrifuged, washed 3× in PBS (to drop concentration of antibiotic below MIC), and resuspended in PBS. The samples were serially diluted in PBS, and 10 µL spots were plated on LB-agar (*E. coli*) or TSB-agar (*S. epidermidis*). Following static incubation for 16 h at 37 °C, persisters were enumerated by counting CFUs. Colony counts between 10 and 100 were used. Percent survival against an untreated control were plotted. Data points represent three replicate experiments, and error bars represent standard error of the mean.

TTO is a mixture that comprises several components. A gas chromatography–mass spectrometry (GC–MS) analysis of the chemical composition of the TTO used in this study was provided by the supplier. The compositions results are shown below in Table S1.

Table S1. Chemical Composition of Tea Tree Essential Oil Provided by the Supplier

**Component Identification**	**%[1]**
Ethanol	0.12
Isobutyral	0.03
Ethyl acetate	0.01
Isobutanol	tr
Isovaleral	tr
2-Methylbutyral	0.02
Isoamyl alcohol	tr
2-Methylbutanol	tr
(3Z)-Hexenol	0.03
Hexanol	0.01
α-Thujene	0.85
α-Pinene	2.40
Camphene	0.01
α-Fenchene	tr
β-Pinene	0.70
Sabinene	0.10
3-Methyl-3-cyclohexenone	0.01
Myrcene	0.80
Pseudolimonene	0.01
α-Phellandrene	0.44
(3Z)-Hexenyl acetate	0.01
α-Terpinene	9.12
Carvomenthene	0.01
Para-Cymene	2.56
Limonene	0.83
1,8-Cineole	2.28
β-Phellandrene	0.76
(Z)-β-Ocimene	tr
(E)-β-Ocimene	0.02
γ-Terpinene	20.10
cis-Sabinene hydrate	0.04
terpinolene	3.26
para-Cymenene	0.06
trans-Sabinene hydrate	0.03
linalool	0.06
unknown	tr
endo-Fenchol	0.02
cis-para-Menth-2-en-1-ol	0.18

4-Hydroxy-4-methylcyclohex-2-enone	0.02
Cosmene isomer l	0.02
trans-para-Menth-2-en-1-ol	0.10
Camphene hydrate	0.01
Unknown	0.03
δ-Terpineol	0.01
Terpinen-4-ol	42.59
Dill ether	0.01
para-Cymen-8-ol	0.09
α-Terpineol	2.95
cis-Piperitol	0.05
trans-Piperitol	0.12
endo-Fenchyl acetate	0.01
exo-2-Hydroxycineole	0.02
Nerol	0.02
Unknown	0.02
Piperitone	0.04
cis-Carvenone oxide	0.01
trans-Ascaridole glycol	0.05
cis-Ascaridole glycol	0.05
Thymol	0.02
Carvacrol	0.02
Unknown	0.05
Bicylcoelemene	0.01
α-Cubebene	0.06
Isoledene	0.06
α-Copaene	0.10
7-Cubebene	0.05
7-Cubebene epimer	0.02
β-Cubebene	0.02
β-Elemene	0.02
Unknown	0.02
α-Gurjunene	0.31
Methyleugenol	0.03
β-Maaliene	0.02
β-Caryophyllene	0.32
γ-Maaliene	0.07
α-Maaliene	0.07
Aromadendrene	1.03
Selina-5,11-diene	0.13
trans-Muurola-3,5-diene	0.10
α-Humulene	0.08
allo-Aromadendrene	0.46
Valerena-4,7(11)-diene	0.04
γ-Gurjunene	0.05

trans-Cadina-1(6),4-diene	0.28
Selina-4,11-diene	0.03
γ-Muurolene	0.01
Germacrene D	0.01
β-Selinene	0.09
allo-Aromadendr-9-ene	0.09
trans-Muurola-4(15),5-diene	0.06
δ-Selinene	0.10
α-Selinene	0.10
Bicyclogermacrene	0.41
Viridiflorene	0.86
Epizonarene	0.01
α-Muurolene	0.14
γ-Cadinene	0.04
trans-Calamenene	0.10
δ-Cadinene	0.95
Zonarene	0.22
trans-Cadina-1,4-diene	0.16
α-Calacorene	0.02
Epiglobulol	0.06
Eudesma-5,7(11)-diene	0.01
Maaliol	0.03
Unknown	0.01
Palustrol	0.05
Unknown	0.01
Spathulenol	0.07
Globulol	0.24
Gleenol	0.02
Viridiflorol	0.13
Cubeban-11-ol	0.10
Ledol	0.04
Eudesm-5-en-11-ol analogue	0.07
Eudesm-5-en-11-ol	0.01
10-epi-Cubenol	0.01
Rosifoliol	0.10
1-epi-Cubenol	0.14
Isospathulenol	0.05
Cubenol	0.08
α-Muurolol	0.03
α-Cadinol	0.01

[1]tr: The compound was detected below 0.005% of the total signal.
